# 

**Published:** 1894-02

**Authors:** 


					﻿VOL XIV.
FEBRUARY, 1894.
NO. 2.
JBHE
HOMEOPATHIC pH
A Monthly Journal of Homoeopathic Materia Medica
and Clinical Medicine.
“ He is a freeman whom truth makes free, and all are slaves beside.”
“Seek the Truth: come whence it may, cost what it will.”
EDITED AND PUBLISHED BY
WALTER M. JAMES, M. E>.
PHILADELPHIA :
No. 1125 SPRUCK STREET.
LONE O.JT :
ALFRED HEATH A CO., Sole Agents bob Gbeat Britain,
114 Ebury Street, Eaton Square.
¥g~Yearly Subscription, $2.50, invariably in advance. Single numbers, 95 cts.
Entered at tbe Philadelphia Poat-Office aa Seoood Claa* Mail Matter.
				

